# Intention Tremor Severity Trajectory: Results from a Prospective Longitudinal Study of Essential Tremor

**DOI:** 10.5334/tohm.1141

**Published:** 2026-02-04

**Authors:** Elan D. Louis, Diane S. Berry, Nora C. Hernandez, Ethan Wainman, Ericka D. Carter, Vibhash D. Sharma

**Affiliations:** 1 Department of Neurology, University of Texas Southwestern Medical Center, Dallas, Texas, USA; 2 Peter O’Donnell Jr. Brain Institute, University of Texas Southwestern Medical Center, Dallas, TX, USA

**Keywords:** Essential tremor, Clinical, Intention tremor, Cerebellar, Cerebellum

## Abstract

**Introduction:**

Patients with essential tremor (ET) may exhibit intention tremor (IT), a sign of cerebellar dysfunction. The prevalence of this sign has been established in cross-sectional studies. To date, however, there have been no cohort studies, re-assessing ET cases prospectively, to determine whether the severity of IT increases over time. The fundamental question is whether IT is progressive in ET.

**Methods:**

90 cases enrolled in a prospective, longitudinal study of elders with ET. IT was assessed in each arm during the finger-nose-finger maneuver (10 repetitions per arm) and scored by a movement disorders neurologist on a 3-item scale – 0 (absent), 0.5 (probable), or 1 (definite), with the IT score (sum of IT in both arms) ranging from 0 – 2. Data from four evaluations every 18 months (T1 – T4) over a 4.5-year period were analyzed.

**Results:**

A one-way repeated measures analysis of variance revealed a time effect for IT, indicating significant variance across time in this variable. Paired sample t-tests indicated that the mean IT score at T4 was greater than those at T1, T2 and T3. There was heterogeneity in the sample, with the T4 score being greater than the T1 score in 46 (51.1%) of 90, but not all individuals.

**Conclusion:**

The cerebellar sign, IT, progressively worsened over time in ET. To our knowledge, this is the first demonstration that *any* canonical cerebellar sign gets progressively worse during prospective follow-up of ET. This clinical observation serves to further the links between ET and progressive cerebellar decline.

## Introduction

Patients with essential tremor (ET) may exhibit intention tremor (IT) [1,2,3]. This tremor is clinically and kinematically indistinguishable from that seen in patients with other disorders of cerebellar degeneration [4]. A number of cross-sectional studies have assessed the *prevalence* of IT among patients with ET, demonstrating a prevalence of approximately 40% [5]. In one cross-sectional study of ET patients with a range of different tremor durations (1 decade, 2 decades, 3 decades, etc.), the authors reported that in longer duration-decades, the mean IT score was higher [6]. In a second cross-sectional study of ET patients, there was an association between the IT score and tremor duration [5]. Following ET patients over time clinically suggests that IT presents and worsens with increasing tremor duration, and IT is often a feature of ET patients undergoing surgical treatments for advanced disease [7]. However, there has not been a study of a cohort of ET cases, re-assessed longitudinally and prospectively, to determine whether the severity of IT increases over time. The fundamental question is whether IT is progressive in ET.

Working from a disease model in which ET is regarded as a form of progressive cerebellar dysfunction and cerebellar degeneration [8,9], one would hypothesize that cerebellar signs might increase in severity with time. As IT has canonically been viewed as a cerebellar neurological sign (i.e., a neurological sign resulting from a lesion in the cerebellum) [10,11], and it is quite prevalent in ET [5], we tested the above hypothesis, assessing whether IT is progressive in ET.

### Methods

ET cases were enrolled in an ongoing, prospective, longitudinal study of elders with ET (COGNET; Clinical Pathological Study of Cognitive Impairment in Essential Tremor; National Institutes of Health Award #R01 NS086736), described in detail, including the use of Washington Heights-Inwood Genetic Study of Essential Tremor criteria for ET [12,13]. The Yale University, Columbia University, and University of Texas Southwestern Medical Center Institutional Review Boards approved the study protocol. All cases provided written, informed consent. Evaluations took place at baseline, and again at 18-month intervals. At each evaluation, cases provided demographic and clinical information, and a videotaped neurological examination was performed. On this videotaped neurological examination, the severity of postural tremor (one examination item) and kinetic tremor (five examination items, including spiral drawing) was assessed in each arm by a movement disorders neurologist on a 0, 0.5, 1, 1.5, 2, 3 scale, yielding a Total Tremor Score (TTS) [12]. TTS values range from 0 (low severity) to 36 (high severity). IT was similarly assessed in each arm during the finger-nose-finger maneuver (10 repetitions per arm), and scored on a 3-item scale as either 0 (absent), 0.5 (probable), or 1 (definite), with the IT score (sum of IT in both arms) ranging from 0–2 [12]. The spiral score was the sum of 0–3 spiral ratings from each arm (range = 0–6); the postural tremor score was the sum of 0–3 ratings from each arm (range = 0–6). Data for these analyses were derived from 90 cases who underwent T1–T4 assessments over a 4.5-year period.

Our analyses were designed to assess the IT score and, for contextual comparison, two other common forms of tremor in ET – postural tremor and kinetic tremor (as assessed through the spiral score). Means, standard deviations and proportions were presented (Table 1). A one-way repeated measures analysis of variance (ANOVA) tested for time effects (i.e., whether there was significant variance across time in a score, Table 2). Paired sample t-tests were used to determine whether scores differed between two selected intervals (e.g., T4 vs. T3) (Table 2). We also compared the baseline characteristics of ET cases who were stratified according to the change in IT score between T1 and T4: T1 > T4, T1 = T4, and T1 < T4 (Table 3).

**Table 1:** Baseline Demographic and Clinical Features of 90 ET Cases.


VARIABLE	DATA

Age (years)	77.5 ± 9.1 (range = 58–95)

Female sex	51 (56.7)

Education (years)	16.0 ± 2.5

Family history of ET	43 (47.8)

Duration of tremor (years)	38.7 ± 21.2 (range = 2–86)

Has been prescribed medication for ET	75 (83.3)

IT score	0.89 ± 0.60 (range = 0–2)

Spiral score	4.12 ± 1.27 (1.5–6)

Postural tremor score	2.60 ± 1.34 (0–6)

Total Tremor Score	23.7 ± 5.6 (range = 10.5–35)


Values represent mean ± standard deviation unless specified otherwise. ET = essential tremor, IT = intention tremor.

**Table 2:** MANOVA Models Assessing Time Effects.


CONDITION	T1	T2	T3	T4	MANOVA (TIME EFFECT)

IT score	0.89 ± 0.60	0.86 ± 0.75	0.99 ± 0.52	1.16 ± 0.63 > T3 > T2 > T1	0.001

Spiral score	4.10 ± 1.28	4.34 ± 1.52	4.15 ± 1.12	4.08 ± 1.20	0.088

Postural tremor score	2.62 ± 1.34	2.98 ± 1.40 >T1	2.26 ± 1.47 <T2 <T1	2.53 ± 1.60 <T2	< 0.001


IT = Intention tremor, T = time. >T3 = value significantly greater than the T3 value at p < 0.05 (paired sample-t test). >T2 = value significantly greater than the T2 value at p < 0.05 (paired sample-t test). >T1 = value significantly greater than the T1 value at p < 0.05 (paired sample-t test). <T2 = value significantly less than the T2 value at p < 0.05 (paired sample-t test). <T1 = value significantly less than the T1 value at p < 0.05 (paired sample-t test).

**Table 3:** Baseline Demographic and Clinical Features of ET Cases Stratified into Three Groups Values represent mean ± standard deviation unless specified otherwise.


VARIABLE	T1 > T4^a^	T1 = T4^a^	T1 < T4^a^	SIGNIFICANCE

n	20	24	46	

Age (years)	77.0 ± 10.4	78.4 ± 8.7	77.3 ± 9.0	p = 0.86^b^

Female sex	10 (50.0)	13 (54.2)	28 (60.9)	p = 0.69^c^

Family history of ET	8 (40.0)	11 (45.8)	24 (52.2)	p = 0.65^c^

Duration of tremor (years)	34.2 ± 23.5	38.7 ± 20.2	40.6 ± 20.9	p = 0.54^b^

Has been prescribed medication for ET	15 (75.0)	21 (87.5)	39 (84.8)	p = 0.50^c^

Total Tremor Score	24.3 ± 6.0	23.9 ± 5.8	23.4 ± 5.4	p = 0.84^b^


ET = essential tremor. ^a^ T1 >T4 (IT score at T1 > IT score at T4), T1 = T4 (IT score at T1 = IT score at T4), T1 < T4 (IT score at T1 < IT score at T4). ^b^ One-way ANOVA. ^c^ Chi square test.

## Results

Baseline characteristics of the sample are shown (Table 1). The ANOVA revealed a time effect for IT (Table 2), indicating significant variance across time in this variable. Paired sample t-tests indicated that the mean IT score at T4 was greater than those at T1, T2 and T3 (Table 2). A parallel repeated measures ANOVA similarly indicated a time effect for postural tremor, although paired-sample t tests indicated rising and falling values across time, with the final value at T4 being no different than the value at T1 (Table 2). An ANOVA model indicated no significant time effect for spiral score (Table 2).

It is important to note that on a group level there were time effects with respect to the IT score, but there was heterogeneity in the sample, with the T4 score being greater than the T1 score in 46 (51.1%) individuals, the same in 24 (26.7%) and less in 20 (22.2%). By contrast, the T3 score was greater than the T1 score in 40 (42.2%) individuals and the T2 score was greater than the T1 score in 32 (35.5%) individuals, showing that as time progressed, a larger and larger proportion of individuals had IT scores that were greater than their T1 score.

We also compared the baseline characteristics of ET cases who were stratified according to the change in IT score between T1 and T4: T1 > T4, T1 = T4, and T1 < T4 (Table 3). The three groups did not differ with respect to baseline age, sex, family history of ET, duration of tremor, use of ET medication or TTS (Table 3).

## Discussion

In this prospective cohort of ET patients, the severity of IT increased over time. This observation is consistent with a disease model that posits greater and greater cerebellar physiological dysfunction over time. It is also consistent with a cerebellar degenerative disease model. Furthermore, to our knowledge, this is the first demonstration that *any* canonical cerebellar sign gets progressively worse during the course of prospective follow-up of ET. As IT is present during goal directed movements, which are involved in the successful performance of many activities of daily living, the functional impact of IT is potentially far higher than that of postural tremor, and small changes in the severity of IT could have disproportionately larger impact on functional ability, although this remains to be tested and demonstrated.

Neither the kinetic tremor score (spiral drawing) nor the postural tremor score showed clear worsening in the limited time frame of this study; however, this should not be interpreted to mean that these tremors do not have a cerebellar origin. The time frame of the current study was limited. It is well-known that the rate of progressive increase in simple kinetic tremor severity in ET occurs gradually over time with small average yearly changes. For example, in a study of 116 ET cases, hand-drawn spirals were rated using the 10-point Bain and Findley rating scale [14] at baseline and at one later time point [15]. The Bain and Findley spiral score increased at an average rate of 0.12 ± 0.23 points per year, indicating a slow progression [15]. Similarly, in a study of 39 ET cases enrolled in a brain donation program, simple kinetic and postural tremor severity were measured with a 36-point rating scale, and this worsened at an average rate of 0.64 ± 1.49 points per year [16]. In a study of 44 ET patients who underwent accelerometry every two years, postural tremor was assessed at baseline and at two follow-up assessments, and postural tremor amplitude did not increase significantly over this time period [17].

There was heterogeneity in our sample. Although as a group there was a time effect observed for IT, there were individuals who in the limited time frame of this study did not have observable worsening. Limitations of this study, as noted below, include the limited time frame as well as the use of a simple ordinal scale for IT with a small range.

One issue to consider is the potential confounding effects of changes in ET medication use on differences in IT severity over time. We have previously shown in this cohort that the majority of cases made some change in their daily medication dosage between T1 and T3 [12]. The primary medications used were primidone and propranolol [12]. These analyses also demonstrated a significant increase across time in primidone dosage but no change across time in propranolol dosage [12]. One might expect the dosing of medication to increase rather than diminish over time – as the disease progresses, the tendency would be to increase the dose of medication in response to worsening symptomatology. Hence, it is likely that increase in tremor severity would be driving medication change rather than the converse. More explicitly, given the above data, we do not think that the observed increase in IT severity over time is the result of a decrease in dosing of ET medications.

The study is not without limitations. First, our assessment of IT was based on an ordinal clinical rating scale; use of objective physiological methods would have facilitated greater precision. However, this relative lack of precision makes it even more remarkable that a time-effect was uncovered; the effect is likely to be greater than we estimated. Second, IT was assessed during a single maneuver, which was performed once with each arm at each assessment. Given the propensity of neurological signs to vary in severity from moment to moment and trial to trial, it would have been more optimal to have repeated the maneuver several times and taken the mean IT score. This would have diminished the potential effects of random variation. However, a learning effect could also have entered the paradigm. Third, longer follow-up duration, beyond 4.5 years would add further to our results, as would enrollment of younger ET cases, in providing data that is more broadly generalizable across all age groups. Despite this, statistical significance was achieved with the study’s sample size and follow-up duration, indicating that it was adequate to address our central question. Finally, we did not assess the correlations between IT and other cerebellar signs (e.g., gait ataxia) or abnormalities on cerebellar imaging (e.g., volumetrics). With respect to gait ataxia, to our knowledge, there are no published data on the onset of and evolution of IT relative to gait ataxia in ET. Theoretically, one might posit that the basis of intention tremor in ET is dysfunction and degeneration of the neocerebellum. The pathological seat of gait ataxia in ET is less apparent – whether it is the result of midline cerebellar degeneration or bi-hemispheric degeneration is not fully clear. Furthermore, published data on the temporal evolution of cerebellar degeneration in ET within different cerebellar regions (e.g., neocerebellum vs. midline structures) is also absent. Hence, there are few available data on which to base a hypothesis about the relative temporal onset of IT relative to gait ataxia in ET.

Strengths of the study included the prospective design. Furthermore, we used a standardized assessment.

In summary, the cerebellar sign, IT, progressively worsened over time in an ET cohort. This clinical observation serves to further the links between ET and progressive cerebellar decline.
